# Feasibility of an Online Shared Reading Intervention in Survivors of Gynecological Cancer: Protocol for a Multiple-Baseline Single-Case Study

**DOI:** 10.2196/91900

**Published:** 2026-08-14

**Authors:** Sonia Tomescu-Stachie, Sarah Kirby, Andrew Merwood, Katy Sivyer, Miznah Al-Abbadey

**Affiliations:** 1 University of Southampton, Highfield Campus, University Road, Southampton, England, SO17 1BJ, United Kingdom, 44 07459884917; 2 Department of Psychology, University of Portsmouth, Portsmouth, England, United Kingdom; 3 Portsmouth Hospitals NHS Trust, Portsmouth, England, United Kingdom; 4 Hampshire and Isle of Wight Healthcare NHS Foundation Trust, Isle of Wight, England, United Kingdom

**Keywords:** single-case experimental design, SCED, feasibility study, online shared reading, digital health intervention, gynecological cancers, quality of life, multiple baselines, cancer survivorship, psychological well-being, arts and health

## Abstract

**Background:**

Gynecological cancers pose significant physical, psychological, and social challenges for those affected. Although psychosocial interventions are components of holistic cancer care, access can be limited by specialized practitioner expertise, wait times, gender-biased care, and accessibility constraints. These barriers highlight a recognized need for alternative, timely, and nonclinical approaches that can complement existing services and relieve system pressures.

**Objective:**

The primary study aim is to evaluate the feasibility and acceptability of an online shared reading program as a novel, adjunctive psychosocial intervention for people affected by gynecological cancers. The secondary aim is to explore the intervention’s preliminary individual-level outcomes on psychological distress, well-being, and quality of life.

**Methods:**

This mixed methods feasibility study uses a multiple-baseline single-case design informed by a realist evaluation theoretical framework. Up to 10 participants aged 18 years or older with a primary diagnosis of gynecological cancer will be recruited internationally. The intervention consists of 6 weekly 1-hour literature reading group sessions delivered via Microsoft Teams. Three baseline assessments at −2 weeks, −1 week, and week 0 will establish outcome stability prior to the intervention, followed by midintervention (week 3) and postintervention (week 6) assessments. Outcome measures include the Short Warwick-Edinburgh Mental Wellbeing Scale, the Kessler Psychological Distress Scale, the WHOQOL-BREF social domain subscale, and 2 global quality of life items from the EORTC QLQ-C30. Individual-level change within these measures will be examined using the reliable change index at a threshold of 1.96 or higher, presented as modified Brinley plots. Feasibility will be assessed and summarized descriptively using recruitment and retention rates, session attendance and completion rates, and reasons for dropout. Acceptability will be examined through semistructured interviews of up to 30 minutes conducted by an independent researcher after the intervention. Interview data will be analyzed using reflexive thematic analysis to explore participants’ intervention experience and perceived mechanisms of change.

**Results:**

This feasibility study was funded in October 2022 as part of a PhD scholarship. Recruitment began in November 2025 and continued until March 2026. At the time of manuscript submission in January 2026, a total of 6 people had registered their interest, of whom 2 met the eligibility criteria. The data analysis plan was formulated but not implemented, with the intervention set to begin in May 2026. Findings are expected to be submitted for publication in November 2026.

**Conclusions:**

Potential implications include that the digital, low-intensity, group-based online shared reading format may offer a novel psychosocial support alternative to survivors of gynecological cancer who may be otherwise excluded due to residing in geographically remote areas, financial constraints, or reluctance to engage with formal psychological therapy. If feasible and acceptable, the intervention may contribute to equitable cancer care provision and inform the design of future implementation studies in digital oncology services.

## Introduction

### Background

Gynecological cancers—including malignancies of the cervix, ovaries, uterus, vagina, and vulva—represent a significant health challenge for women and gender-diverse people assigned or presumed female worldwide [1-3]. Prevalence and age incidence vary across settings—cervical cancer disproportionately affects those in low- and middle-income countries largely due to higher human papillomavirus prevalence and limited screening [4,5], whereas endometrial cancer is increasing in high-income regions, linked to obesity and aging populations [6,7]. Even as advances in diagnosis and treatment have improved survival rates, those affected can experience psychological distress, including anxiety, depression, fear of recurrence, anger, shame, low self-esteem, and reduced quality of life (QoL) [3,8], often compounded by disruption to reproductive and sexual health [9,10]. Gynecological cancers also carry gendered stigma, with transgender people experiencing a particular sense of disconnect when they are diagnosed with a cancer that does not align with their gender identity [11,12]. As a result, they often report feeling uncomfortable attending support groups organized according to sex assigned at birth [13,14].

However, despite its recognition as an essential component of cancer care, psychosocial care remains inconsistently embedded worldwide [15,16]. Across both high- and low-income countries, survivorship support is often fragmented, reactive, and primarily biomedical, focusing on disease surveillance rather than long-term emotional and social recovery [17,18]. Existing psychosocial interventions—including counseling, cognitive behavioral therapy, and mindfulness-based approaches—have shown efficacy in reducing anxiety and depression in survivors of cancer [19,20]. However, the evidence base remains heterogeneous, with substantial variation in intervention content, delivery format, and outcome measurement, making it difficult to determine what works best, for whom, and under which circumstances [21]. Structural barriers including workforce shortages, long wait times, and stigma around mental health can further exacerbate inequalities and restrict access [22-24]. Additionally, clinical guidelines [25] offer limited direction on how best to embed psychosocial care across the patient pathway. This creates the need for accessible, scalable, and adjunctive approaches deliverable outside specialist services, particularly for those who fall below the threshold for formal psychological support.

One promising strategy is using creative arts and literature–based digital health interventions (hereafter referred to as “arts and health”) as a form of community psychosocial support [26,27]. Their online format can improve accessibility for people living in remote or underserved areas and lessen the stigma associated with formal psychological therapy [28,29]. Additionally, arts and health interventions remain relatively low cost, resource efficient, and scalable to diverse populations [30,31]. Within this field, shared reading (SR) is an established model of facilitated literary engagement. It involves trained reader leaders who select short stories, novel extracts, or poems to read aloud. During the reading, planned reflective pauses are used to invite participants’ thoughts, feelings, and associations around classic literature [26,32-34]. This deliberate use of meaningful pauses is a defining feature of SR intended to foster deep engagement with the material read and support immediate, in-the-moment reflections [35]. Studies of SR across diverse populations, including prisons, dementia care, substance use recovery, and chronic pain, report improvements in psychological well-being, emotional regulation, and self-awareness [36-38]. These benefits are perhaps reflective of the nonclinical settings in which the intervention takes place—often a library, community center, or virtual room—where emotional expression and a sense of belonging are created via the collective experience of literature reading. Notably, the values that underpin SR also closely align with trauma-informed frameworks, building around safety, choice, collaboration, trust, and empowerment as core principles of care [39,40]. In SR, participants can speak or stay silent, engage with the text directly or through its characters, and decide how much of their own story to bring into the room. These features make SR a conceptually strong strategy for adaptation to people living with and beyond gynecological cancer, for whom issues of control, identity, and safety are often central [41,42].

However, there is still uncertainty around the methodological and evaluative value of arts and health interventions due to poor study design, insufficient focus on feasibility studies, and a lack of consensus on how to measure their value [43,44]. Recommendations include incorporating mixed methods research to capture both the qualitative and quantitative impacts of arts and health interventions [45,46]. Feasibility and pilot studies are also thought of as foundations for later randomized controlled trials, providing critical evidence on design optimization, resource needs, and recruitment strategies [47,48]. Such preparatory work can offer insights often missing from efficacy trials and could inform policy, funding decisions, and the justification for larger-scale implementation.

Consistent with the Medical Research Council’s [49,50] guidance on explaining *why* an intervention works rather than exclusively on *if* it does, this feasibility study is underpinned by a realist evaluation (RE) approach [51]. RE seeks to explore what works, for whom, and in what circumstances through context-mechanism-outcome configurations. Specifically, “context” refers to the conditions under which the online SR may work, such as illness stage, prior interest in literature, or openness to discussing emotions. “Mechanism” relates to the intervention processes that may drive change, including emotional resonance with the literature or others in the group, self-reflection, or narrative reappraisal. Finally, “outcome” refers to the benefits that online SR may produce, including reduced distress; improved well-being or QoL; and feasibility measures such as attendance, engagement, and acceptability. Importantly, RE complements the study’s rationale because it recognizes that the intervention may be meaningful for some participants and less so for others depending on their unique cancer experiences, needs, and readiness to engage.

### Study Objectives

The primary study objective is to assess the feasibility and acceptability of a 6-week online SR intervention for women and gender-diverse people affected by gynecological cancers. The secondary objective is to explore the intervention’s preliminary individual-level effects on psychological well-being, distress, and QoL.

## Methods

### Intervention Development and Content

The intervention draws on the established face-to-face SR model developed by The Reader [35] but takes place within a virtual Microsoft Teams environment. Specifically, the intervention development followed 3 sequential steps: first, the lead author (ST-S) completed the mandatory 3-week *Read to Lead* facilitator program created by The Reader [35]. Training included guidance on facilitating open-ended literary discussions, using reflective pauses and questions, and maintaining respect and safety within a group environment. The latter aspect involves reiterating, at the start of each week, that sessions are not recorded and that any data handling will comply with the UK Data Protection Act 2018 and the General Data Protection Regulation.

Second, all selected literature was chosen either from The Reader’s anthology bank or in consultation with The Reader [35] (Multimedia Appendix 1). Texts with explicit references to cancer, illness, or death were excluded to avoid inadvertently influencing the content of the discussions or participants’ emotional responses.

Third, the digital adaptation of the sessions was refined in collaboration with and in response to feedback from a 1-hour patient and public involvement and engagement (PPIE) group; this informed both the content and online delivery format of the intervention. Specifically, in May 2024, a total of 6 community members were recruited via social media and asked to review and provide feedback on study materials and content and the appropriateness of online vs face-to-face delivery. Participants discussed the potential benefits of online sessions, including the lessening of cost and time burdens associated with traveling, as well as the potential widening of participation nationally and internationally. One PPIE participant was recruited to provide ongoing input throughout the research process. Their role includes supporting with participant recruitment, reflecting on qualitative data interpretation, and collaborating on the dissemination of findings. All contributions are reimbursed in line with National Institute for Health and Care Research guidance of £25 per person per hour (£1=US $1.35 as of August 7, 2026).

The finalized intervention model will consist of 6 weekly group sessions, each lasting 1 hour and delivered via Microsoft Teams. Sessions will be scheduled on the same day and time each week, to be confirmed with participants prior to intervention start. This decision will support a sense of continuity and allow participants to plan around existing commitments. Additionally, to preserve spontaneity, texts will be shared with participants via email 15 minutes before each session. Participants will be required to keep their cameras on to maintain a sense of group presence and connection; however, they will be free to contribute to discussions either verbally or via the chat function. Texts will be screen shared during sessions to ensure that all participants can easily follow along, thereby preserving the live, interactive presence of the literature. Session format and structure is shown in Table 1. Additionally, a TIDieR (Template for Intervention Description and Replication) checklist is provided as Checklist 1.

**Table 1.** Online shared reading session structure repeated each session^a^.

Session components	Description
Review rules of engagement (approximately 10 min)	Study introduction (ie, what shared reading is; sessions take place every week for 6 wk; duration of between 60 and 90 min; scheduled at the same time and day of the week) Confidentiality agreement (ie, data handling; GDPR^b^; sessions are not recorded) Microsoft Teams tutorial covering essential platform features (audio and video settings and raising their hand or unmuting to speak; option to use the chat function) Agreement to have cameras on
Reading aloud (approximately 5-10 min)	Reader leader reads the opening lines, a section, or the full text (ie, a poem) aloud
Pause and reflect (approximately 15 min)	Silence held to allow for responses from the group by asking whether there are any thoughts or reflections on what was read
Participants invited to read (approximately 5-10 min)	Participants are asked whether they would volunteer to reread lines or passages aloud in their own voice and style.
Group discussion (approximately 15 min)	Facilitated by the reader leader, participants share thoughts, reflections, and responses (ie, which line or image felt easiest to picture and why, what feeling the poem left behind, which part felt most familiar or surprising, which line they would want to discuss further, and why they thought the author used those visual images to make their mark)

a Consideration of backup literature and session timings: if participants struggle to engage, a backup text is introduced, and the process is repeated from step 2.

b GDPR: General Data Protection Regulation.

### Participants

Up to 10 women and gender-diverse people aged 18 years or older diagnosed with a primary form of gynecological cancer will be invited to participate in the intervention. To be eligible, participants must not be receiving other psychological interventions at the time of recruitment. This exclusion reflects the potential for concurrent specialized therapies to confound interpretation of online SR’s preliminary individual-level effects on psychological distress, well-being, and QoL. The full eligibility criteria are shown in Textbox 1.

Textbox 1. **Feasibility study inclusion and exclusion criteria.**

**Inclusion criteria**

- Women and gender-diverse people aged 18 y or older
- Gynecological cancer (ie, cervical, ovarian, uterine, vaginal, vulvar, or fallopian tube) as primary diagnosis
- Either in active treatment or in remission

**Exclusion criteria**

- Current or previous participation in shared reading specifically
- Women and gender-diverse people with cancer of unknown primary or gynecological cancer as secondary (ie, affected by multiple cancers, but primary cancer is unknown)
- Current receipt of other psychological interventions (ie, counseling or cognitive behavioral therapy)
- Inability to comprehend the study and/or provide informed consent (ie, insufficient command of the English language in the absence of someone who can adequately interpret)

As this is not a full-scale trial, nor is the primary aim to evaluate the effectiveness of online SR, a formal sample size calculation was not required [52,53]. Instead, the target sample was determined based on 2 factors. First, single-case experimental design (SCED) methodology requires a minimum of 3 participants to demonstrate an experimental effect [54]. Second, the traditional SR model typically consists of 2 to 12 attendees [32]. Consequently, this feasibility study will aim to recruit between 4 and 10 participants and will not be designed or powered to evaluate the effectiveness of online SR; the absence of a comparator condition and the small sample size will preclude inferential conclusions about efficacy.

### Recruitment

International study recruitment is planned from November 2025 to March 2026. Potential participants will be identified through clinician referral at patient identification centers within the South Central Regional Research Delivery Network and via study posters displayed in oncology waiting rooms. If required to meet recruitment targets, the study will also be promoted internationally, via cancer charities and social media platforms (ie, LinkedIn, X [formerly known as Twitter], and Facebook). Interested parties will contact the lead author (ST-S) directly and will be asked to complete a consent form and a screening and demographics questionnaire, as well as 3 weekly staggered baseline outcome measures (Multimedia Appendix 2). These data points will be collected via Qualtrics (Qualtrics International Inc), the same online platform where participants will indicate their availability for taking part in the online SR intervention.

### Study Design

The study uses a mixed methods, nonrandomized, multiple-baseline, within-subject design consistent with SCED methodology. The multiple baselines will be operationalized via 3 repeated preintervention assessments (at −2 weeks, −1 week, and week 0) to establish outcome measure stability. Afterward, participants will commence the 6-week intervention simultaneously. Participant or researcher blinding is not possible. Data collection points are shown in Table 2.

**Table 2.** Data collection points.

	Screening (–4 wk)	Baseline 1 (–2 wk)	Baseline 2 (–1 wk)	Baseline 3 (0 wk)	Midintervention time point (3 wk)	Postintervention time point (6 wk)
Demographics (ie, age, gender, ethnicity, educational level, employment status, and treatment status [ie, in treatment or in remission or cancer free])	✓	✓				
Psychological well-being (SWEMWBS^a^) [55]		✓	✓	✓	✓	✓
Psychological distress (K10^b^) [56]		✓	✓	✓	✓	✓
World Health Organization quality of life instrument social domain subscale [57]		✓	✓	✓	✓	✓
Two global items (health and QoL^c^) from the EORTC QLQ-30^d^ [58]		✓	✓	✓	✓	✓
Exit survey (for dropout participants; Multimedia Appendix 3)—to be administered at any exit point						

a SWEMWBS: Short Warwick-Edinburgh Mental Wellbeing Scale.

b K10: Kessler Psychological Distress Scale.

c QoL: World Health Organization Quality of Life social domain subscale.

d EORTC QLQ-C30: European Organisation for Research and Treatment of Cancer Quality of Life Questionnaire Core 30.

### Data Collection

All questionnaire data will be collected via Qualtrics. Following submission of the consent form, participants will automatically receive a copy via the platform; copies may also be requested directly from the lead author via email. Participants will be assigned unique identification numbers at enrollment; data will be stored on password-protected university servers and handled in accordance with institutional data governance procedures.

Prior to the start of the online SR intervention, participants who require support with the Microsoft Teams platform will be offered an optional 15-minute tutorial covering how to join a meeting, use the chat and hand-raising functions, and manage audio and video settings. The tutorial will be delivered at a time convenient to each participant.

### Outcome Measures

#### Feasibility and Acceptability

Feasibility will be assessed through recruitment ease and retention rates—including the number of eligible individuals expressing interest relative to the final sample, the time needed to finalize recruitment, the reasons for declining participation or dropping out, and session attendance rates. Acceptability will be explored through postintervention semistructured interviews lasting up to 30 minutes conducted online via Microsoft Teams by a researcher independent to the study team. Verbal informed consent will be obtained and audio recorded prior to the interviews, with timings arranged to accommodate individual preferences, appointment commitments, energy levels, and interviewer commitments. The interview guide can be found in Multimedia Appendix 4.

Intervention fidelity will be assessed using the National Institutes of Health Behavior Change Consortium framework [59], or an equivalent fidelity model, to evaluate adherence, exposure, quality of delivery, participant responsiveness, and the extent to which the intervention was delivered as intended.

#### Individual-Level Preliminary Outcomes

Preliminary individual-level outcomes will be assessed using the Short Warwick-Edinburgh Mental Wellbeing Scale [55], the Kessler Psychological Distress Scale [56], the social domain subscale of the World Health Organization QoL instrument [57], and 2 global QoL items from the European Organisation for Research and Treatment of Cancer Quality of Life Questionnaire Core 30 [58].

### Withdrawal and Dropout

Participation is entirely voluntary, and participants are free to withdraw their consent at any point without needing to provide a reason; doing so will not impact their care or legal rights. If a participant who initially gave informed consent loses the ability to consent during the study (ie, due to progression of their illness), they will be withdrawn from the research. However, any data collected before the loss of capacity will be retained and included in the study unless the participant specifically requests complete data removal.

Withdrawal may take 2 forms: participants may inform the lead author (ST-S) of their withdrawal, with the option to either (1) cease all participation, including the intervention, questionnaires, exit survey, and interview; or (2) withdraw from the intervention only while continuing to complete questionnaires and/or the exit survey and/or the interview. Alternatively, participants may withdraw without notification; in this case, the lead author will attempt follow-up contact up to 2 times via email. An exit survey will be administered where possible to all participants who withdraw at whatever point withdrawal occurs to capture dropout reasons.

### Data Analysis Plan

#### Feasibility Measures

Recruitment, attrition, and questionnaire completion rates will be reported descriptively, including 95% CIs where applicable, alongside any observed floor and ceiling effects. Descriptive statistics will include means or medians, SDs or IQRs, frequencies, and proportions as appropriate for the distribution of data.

#### Preliminary Quantitative Outcomes

As this is a feasibility study not powered for inferential testing, preliminary outcomes will be examined descriptively and visually. The reliable change index will be calculated for each outcome measure (Short Warwick-Edinburgh Mental Wellbeing Scale, Kessler Psychological Distress Scale, World Health Organization QoL instrument social domain subscale, and the 2 European Organisation for Research and Treatment of Cancer Quality of Life Questionnaire Core 30 global items). A value of 1.96 or higher will indicate statistically reliable change at the 95% CI, and data will be presented as modified Brinley plots across the 5 time points. This will enable a visual representation of the data across 3 baselines and the midintervention and postintervention time points, consistent with established graphical analysis methods for SCED [60]. Missing data will be handled using a complete-case approach, with the extent and pattern of missing data reported descriptively, as recommended for feasibility studies of this scale [61,62]. As a sensitivity check, outcomes will be examined for participants meeting the minimum attendance threshold (≥3 sessions) compared with the full attendance sample.

#### Qualitative Analysis

Verbatim transcripts from audio-recorded interviews will be analyzed using the 6-phase approach of reflexive thematic analysis [63,64]. Themes will be developed inductively, with attention to both semantic and latent levels of meaning, and interpreted through the lens of the RE framework [51] to explore context-mechanism-outcome configurations relevant to participants’ experiences of the online SR intervention. The NVivo software (version 20; Lumivero) will enable data organization.

To support reflexivity, the lead author (ST-S) will maintain an analytic journal to document decisions made during online SR sessions, coding, and theme development. Following guidance on “becoming a knowing researcher” [65], their position will be that of conscious engagement with their own assumptions, values, and research role. This process will aid in explicitly reflecting on how their positionality as both researcher and facilitator (ie, White, educated female with no lived experience of gynecological cancer and with previous experience of running SR groups) may have shaped sessions, interview questions, and analyses. Reflexive dialogue with supervisors and PPIE collaborators will further support this process by introducing critical perspectives that challenge implicit assumptions and the idea that bias should or even could be eliminated.

### Integration of Quantitative and Qualitative Findings

Quantitative and qualitative findings will be integrated to provide a comprehensive understanding of the intervention’s impact. Qualitative data will be used to clarify or expand upon the quantitative results within the RE framework [51]. Analyses will first be considered separately, with careful attention paid to how the findings align or diverge when forming overall conclusions.

### Ethical Considerations

Ethics approval was obtained from the University of Southampton (reference 101967) and the National Health Service Health Research Authority (reference 25/PR/0739), with study procedures to be conducted in accordance with the Declaration of Helsinki. Informed consent will be voluntary and ongoing, where participants may withdraw at any time without penalty or disruption to their care. Written consent will be obtained from all participants prior to enrollment via Qualtrics. Verbal informed consent will be collected and audio recorded via Microsoft Teams before the start of each interview. To thank participants for their time, a £50 Amazon voucher will be offered to those who attend at least 3 of 6 sessions, allowing participants several absences to account for potential illness, hospital appointments, or unforeseen circumstances.

Clinical safety will be monitored by clinical oncologists, cancer nurses, and care providers as part of the participants’ usual care. The lead author will be supervised by a clinical psychologist (AM), 2 health psychologists (MA-A and SK), and an academic supervisor (KS) throughout the study duration. If participants become distressed during the sessions, the lead author (ST-S) will attempt to first discuss their thoughts and feelings in the session. If participants decline, they will be offered a debrief after the end of the session, and the lead author will check in with them via email after the session. At the end of the online SR intervention, participants will automatically be offered signposting information to the Macmillan Information and Support Centre and their cancer clinical nurse specialist following the model of psychological care outlined in the National Institute for Health and Care Excellence guidelines [25]. Any instances of psychological distress will also be discussed in supervision with the team.

## Results

**Figure 1.**
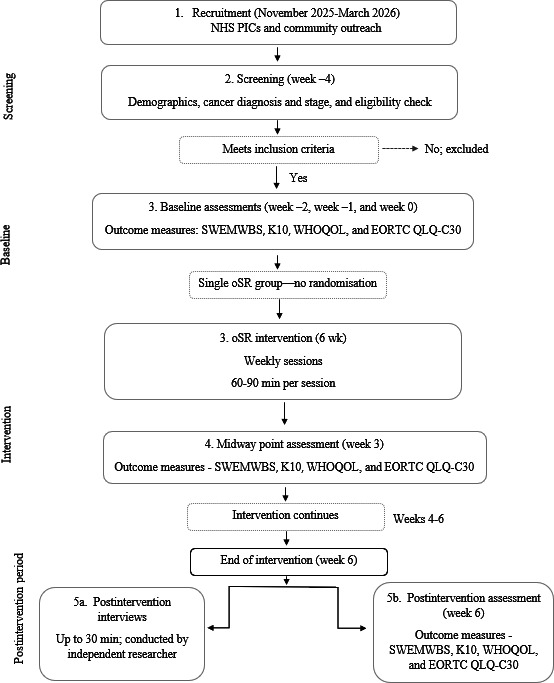
Flowchart of the study timeline. EORTC QLQ-C30: European Organisation for Research and Treatment of Cancer Quality of Life Questionnaire Core 30; K10: Kessler Psychological Distress Scale; NHS: National Health Service; oSR: online shared reading; PIC: patient identification center; SWEMWBS: Short Warwick-Edinburgh Mental Wellbeing Scale; WHOQOL: World Health Organization quality of life instrument.

This feasibility study received funding in October 2022 as part of a PhD scholarship. Recruitment started in November 2025 and continued until March 2026. At the time of manuscript submission in January 2026, a total of 6 individuals had registered their interest, of whom 2 met the eligibility criteria. The data analysis plan was developed in January 2025 to facilitate university and National Health Service Health Research Authority ethical submissions and registered on the Open Science Framework in May 2025 [66]. The analysis has not yet been implemented, with the intervention commencing in May 2026. Dissemination of findings is planned for November 2026 and will follow SCRIBE (Single-Case Reporting Guideline in Behavioral Interventions) guidelines [67]. The study timeline is summarized in Figure 1.

Research findings will be published in a peer-reviewed journal to ensure rigorous academic scrutiny and broad dissemination. Target journals will be selected collaboratively with the research team based on their relevance to oncology and arts-based intervention studies. Results may be presented at relevant national and international conferences to engage with the academic and clinical community, fostering discussions and potential collaborations. Findings may also be shared at charity events and forums related to gynecological cancers to raise awareness, support advocacy efforts, and reach a wider audience beyond the academic sphere. The results will form the basis for completion of a PhD thesis to be submitted to the University of Southampton by the end of 2026.

## Discussion

### Expected Findings

This study aims to evaluate a 6-week online SR intervention designed for women and gender-diverse people affected by gynecological cancers. Potential implications include that the digital, low-intensity, group-based online SR format may offer a novel mechanism to reach survivors who may be otherwise excluded due to residing in geographically remote areas, gender-biased care, financial constraints, and inability or reluctance to engage with formal psychological therapy. Demonstrating potential benefits could also justify future large-scale trials and inform service commissioning decisions, aligning with calls for early-phase experimental designs to bridge the gap between innovation and implementation [47,48].

### Strengths and Limitations

The study’s use of a multiple-baseline SCED enables in-depth individual-level analysis despite the small sample size. Additionally, the study uses a mixed methods approach alongside an RE framework [51] to explore not only whether online SR is a feasible and acceptable intervention but also for whom and under what conditions. Finally, the intervention development was informed by PPIE from design to dissemination, ensuring that the online SR model was shaped alongside the people it aims to support.

However, consideration must be given to issues of equity and inclusivity in the design and delivery of the online SR intervention, including potential limitations in accessibility for individuals with lower digital literacy, limited educational opportunities, or neurodivergence and specific learning difficulties. Such factors may risk inadvertently excluding those who could benefit most from community-based psychosocial interventions. However, SR is intentionally designed to be open and participatory, accommodating a wide range of abilities and preferences; sessions emphasize listening as much as reading, and there is no expectation for participants to interpret texts in a particular way or even verbally contribute. This flexibility will allow individuals to engage with online SR at their own comfort level, supporting inclusion while preserving online SR’s ethos of shared, reflective connection through literature. Future refinement could explore the barriers to engagement among people who decline or withdraw from the online SR intervention to better understand their motivations and inform strategies to improve uptake and retention.

### Conclusions

This feasibility study will be the first to examine online SR as an adjunctive psychosocial intervention for people affected by gynecological cancers. In doing so, the proposed intervention responds to a recognized gap in accessible, low-intensity psychosocial support for this population. Importantly, it may offer community-based peer support for those managing emotional distress who may prefer not to engage with or lack access to formal therapeutic support.

While the study is exploratory, it will offer foundational data on the acceptability, feasibility, and preliminary efficacy of online SR. Additionally, it will inform the potential intervention development components of a future, adequately powered, full-scale trial. Should the study demonstrate online SR to be feasible and acceptable, implications include having a scalable, low-cost model that could be embedded alongside existing survivorship pathways. Finally, its fully online delivery has potential to reach those facing geographical, financial, or psychological barriers to formal care, supporting more equitable access across diverse health system settings.

## Supplementary material

10.2196/91900 Multimedia Appendix 1 Online shared reading intervention content.

10.2196/91900 Multimedia Appendix 2 Screening and demographics questionnaires.

10.2196/91900 Multimedia Appendix 3 Exit survey.

10.2196/91900 Multimedia Appendix 4 Interview schedule.

10.2196/91900 Checklist 1 TIDieR checklist.

## Abbreviations

PPIE: patient and public involvement and engagement
QoL: quality of life
RE: realist evaluation
SCED: single-case experimental design
SCRIBE: Single-Case Reporting Guideline in Behavioral Interventions
SR: shared reading
TIDieR: Template for Intervention Description and Replication
