# Supplementary material for: Feasibility of an Online Shared Reading Intervention in Survivors of Gynecological Cancer: Protocol for a Multiple-Baseline Single-Case Study
Source: JMIR Res Protoc. 2026 Aug 14;15:e91900. doi: 10.2196/91900 (PMC13475789; doi:10.2196/91900)
Supplement: Supplementary file 1 [file resprot-v15-e91900-s001.docx]

**Supplementary Material 1**

online Shared Reading (oSR) Session Structure

| Session number | Proposed literature |
| --- | --- |
| Session 1 | **Main poems:**   - Wild Geese, by Mary Oliver - Ambition’s Trail, by Ella Wheeler Wilcox     ***Backup:***   - Happy the Man, by John Dryden - I Choose the Mountain, by Simon Howard |
| Session 2 | **Main short story:**   - Breaking the Pig, by Etgar Keret   ***Backup:***   - [The Voyage](https://www.eastoftheweb.com/short-stories/UBooks/Voya.shtml), by Katherine Mansfield |
| Session 3 | **Main book**:   - [Anxious People](https://www.readinggroupguides.com/reviews/anxious-people/excerpt), by Fredrik Backman (chapters 1 & 15)   ***Backups:***   - Lines, by Thomas Hood - Love, by George Herbert |
| Session 4 | **Main poems:**   - Daffodils, by William Wordsworth - A Girl’s Reverie, by Ella Wheeler Wilcox   ***Backup:***   - A man called Ove, by Fredrik Backman (chapter 2) |
| Session 5 | **Main book**:   - Great Expectations, by Charles Dickens (chapter 8)   ***Backups:***   - Little Women, by Louisa May Alcott (chapter 1) - The Secret Garden, by Frances Hodgson Burnett (chapter 21) |
| Session 6 | **Main novel:**   - Still, I Rise by Maya Angelou - The Guest House by Mewlana Jalaluddin Rumi - There will come Soft Rain by Sara Teasdale     ***Backups:***   - The Gift of the Magi, by O’Henry - Sonnet 29, by William Shakespeare |
