# Supplementary material for: Feasibility of an Online Shared Reading Intervention in Survivors of Gynecological Cancer: Protocol for a Multiple-Baseline Single-Case Study
Source: JMIR Res Protoc. 2026 Aug 14;15:e91900. doi: 10.2196/91900 (PMC13475789; doi:10.2196/91900)
Supplement: Supplementary file 2 [file resprot-v15-e91900-s002.docx]

**Multimedia Appendix 3**

## Qualtrics Screening and Demographics Questionnaires

**Screening**

1. What is your age? Please specify below:

Cancer-specific characteristics:

1. Are you currently undergoing treatment for gynaecological cancer?

If no -> how long have you been in-remission/cancer-free?

2. What gynaecological cancer were/are you affected by?
3. Are you currently receiving any psychological treatment (e.g., counselling, CBT)?

-> If yes, please specify

-> No

4. Have you participated in any [Shared Reading](https://www.thereader.org.uk/what-we-do/shared-reading/) before? (this is specific type of group, not the same as a book club or reading group)

-> Yes

-> No

5. If you wish to participate in Shared Reading, then please add your email in the box below [open-text box]

5a. If you wish to participate, what is your motivation for doing so? [open-text box]

5b. If you do not wish to participate, then please could you tell us why? [open-text box]

## **Demographic**

1. What gender do you identify as?
   a. Female
   b. Transgender
   c. Non-binary
   d. Prefer not to say
2. What is your ethnicity? Please specify below:
3. What is the highest degree or level of school you have completed?
   a. Some high school, no diploma
   b. High school/college graduate or equivalent
   c. Bachelor’s degree (e.g. BA, BSc)
   d. Master’s degree
   e. Doctorate (e.g. PhD, EdD)
   f. Other (please specify) _____
4. What is your current employment status?
   a. Employed (full-time)
   b. Employed (part-time)
   c. Unemployed (currently looking for job)
   d. Unemployed (currently not looking job)
   e. Student
   f. Retired
   g. Self-employed
   h. Other (please specify): _____
6. Can you recall when you were first diagnosed?
7. Have you experienced a recurrence?

**->**If yes, please specify.

**->** No

8. Do you suffer from any other health condition?

**->** If yes, please specify.

**->** No
