# Supplementary material for: Feasibility of an Online Shared Reading Intervention in Survivors of Gynecological Cancer: Protocol for a Multiple-Baseline Single-Case Study
Source: JMIR Res Protoc. 2026 Aug 14;15:e91900. doi: 10.2196/91900 (PMC13475789; doi:10.2196/91900)
Supplement: Supplementary file 3 [file resprot-v15-e91900-s003.docx]

**Multimedia Appendix 4**

Exit Survey (participant drop-out)

1) What was the primary reason you did not complete all six oSR reading sessions? 
*(Select all that apply)*

☐ Time constraints

☐ Technical difficulties (e.g., internet issues, trouble accessing Zoom/Teams)

☐ Loss of interest in the sessions

☐ Content was not relevant or engaging

☐ Emotional difficulties (e.g., too distressing, overwhelming)

☐ Health-related challenges (e.g., fatigue, illness, recurrence)

☐ Other (please specify): [open text box]

2) How easy or difficult was it to attend the oSR sessions each week? 
*(Likert scale: 1 = Very Difficult 5 = Very Easy)*

☐ 1 Very Difficult

☐ 2 Difficult

☐ 3 Neutral

☐ 4 Easy

☐ 5 Very Easy

3) To what extent did the prospect of a £50 voucher influence your decision to attend at least half of the sessions?

☐ 1 Not at all

☐ 2 Slightly

☐ 3 Neutral

☐ 4 Moderately

☐ 5 A great deal

4) Did you feel the oSR sessions met your expectations? Why or why not? 
*(Open text box)*

5) Would you consider rejoining an oSR reading group, in the future, if adjustments were made? 
*(Likert scale: 1 = Not at all, 5 = Definitely)*

☐ 1 Not at all

☐ 2 Unlikely

☐ 3 Unsure

☐ 4 Likely

☐ 5 Definitely

6) What changes or improvements would have made it easier for you to continue participating? 
*(Open text box)*
