# Supplementary material for: Feasibility of an Online Shared Reading Intervention in Survivors of Gynecological Cancer: Protocol for a Multiple-Baseline Single-Case Study
Source: JMIR Res Protoc. 2026 Aug 14;15:e91900. doi: 10.2196/91900 (PMC13475789; doi:10.2196/91900)
Supplement: Supplementary file 4 [file resprot-v15-e91900-s004.docx]

**Multimedia Appendix 5**

Qualitative Interview Guide

**Before the Interview:**

- Verbal explanation of project and process
- Written information given (PIS)
- Audio record consent

**Section 1 (expectations)**

1. Before joining, what did you know about Shared Reading groups?

- Have you ever attended reading groups or book clubs before?
- If so, how did those compare to oSR?

2. What were your initial expectations about the oSR sessions?
   - What did you think the sessions would be like?
   - Did you have any concerns before starting?
   - If so, what were they?

**Section 2 (experience & engagement)**

3. Could you describe what it was like to take part in the sessions?
   - Did your experience change over time?
4. Were there any moments when you felt particularly engaged during the sessions?

- If yes, what do you think contributed to this?

5. Were there any moments where you felt less engaged or found it difficult to participate?
   - What do you think played a role?
6. To what extent did the £50 voucher influence your decision to attend at least half of the sessions?

**Section 3 (self-perceived impact)**

7. Have you noticed any changes in yourself because of the sessions?

- If so, what kinds of changes? (e.g., emotional, social)

8. Did the sessions affect how you reflect on your experiences of cancer?
   - If so, could you describe how?
9. To what extend did the session impact any sense of connecting to others?
   - If so, within or beyond the group?

**Section 4: Accessibility & practical considerations**

10. How do you feel about attending and engaging with the sessions online?
    - Did the online format support or hinder your engagement?
11. Were there any factors (e.g., technical issues, personal circumstances, emotional readiness) that made participation difficult?
12. Were there any factors that made it easier for you to participate?

**Section 5: Improvements & future considerations**

13. If the sessions were to be improved, what suggestions would you make?
14. Do you think oSR should be offered free within the NHS?
    - Why or why not?
15. Is there anything else you would like to share about your experience with oSR?

*Please note that these questions serve as a flexible guide for the interviews. They may be refined. This ensures that the interview approach remains responsive to participant’s lived experiences and priorities.*
