# Supplementary material for: Feasibility of an Online Shared Reading Intervention in Survivors of Gynecological Cancer: Protocol for a Multiple-Baseline Single-Case Study
Source: JMIR Res Protoc. 2026 Aug 14;15:e91900. doi: 10.2196/91900 (PMC13475789; doi:10.2196/91900)
Supplement: Supplementary file 5 [file resprot-v15-e91900-s005.docx]

**The TIDieR (Template for Intervention Description and Replication) Checklist*:**

Information to include when describing an intervention and the location of the information

| Item number | Item | Where located ** | |
| --- | --- | --- | --- |
|  |  | Primary paper  (page or appendix  number) | Other ^†^ (details) |
|  | **BRIEF NAME**   1. Provide the name or a phrase that describes the intervention | Page 1 | The intervention is described in the title as a “*Feasibility of an Online Shared Reading Intervention in Survivors of Gynaecological Cancer: Protocol for a Multiple-Baseline Single-Case Mixed Methods Study.* |
|  | **WHY**   2. Describe any rationale, theory, or goal of the elements essential to the intervention | Pages 4-7 | Rationale, theory and aims are described in the introduction section. |
|  | **WHAT**   3. Materials: Describe any physical or informational materials used in the intervention, including those provided to participants or used in intervention delivery or in training of intervention providers. Provide information on where the materials can be accessed (e.g. online appendix, URL). | Pages 7-9 | Materials are described on Pages 7 to 9. Any additional material details (i.e., oSR session content) are presented as Supplementary Material 1. |
|  | 4. Procedures: Describe each of the procedures, activities, and/or processes used in the intervention, including any enabling or support activities. | Pages 8-9 | Session format is presented in Table 1, while intervention content is available as supplementary Material 1. |
|  | **WHO PROVIDED**   5. For each category of intervention provider (e.g. psychologist, nursing assistant), describe their expertise, background and any specific training given. | Pages 7 and 17 | The oSR intervention will be delivered by the lead author (ST), who has completed mandatory training as a Reader Leader. ST is a PhD Researcher and Trainee Health Psychologist, bringing a background in both academic research and clinical health psychology training. As part of the broader research team, ST will be supported by a clinical psychologist (AM), two health psychologists (MAA and SK), and a lecturer in clinical psychology (KS), who will provide supervision and guidance as needed. |
|  | **HOW**   6. Describe the modes of delivery (e.g. face-to-face or by some other mechanism, such as internet or telephone) of the intervention and whether it was provided individually or in a group. | Page 12 | online Shared Reading (oSR) group sessions will be delivered solely online, via Microsoft Team, once per week for a total of 6 weeks. They will take place at the same time and day each week, at a time and day agreed – prior to the intervention - with participants. |
|  | **WHERE**   7. Describe the type(s) of location(s) where the intervention occurred, including any necessary infrastructure or relevant features. | Page 12 | Sessions will take place online, via Microsoft Teams. To ensure that participants are comfortable with the technology, the lead author (ST) will offer a 15-minute tutorial on Microsoft Teams features, to be delivered optionally, at the convenience of participants. This tutorial will cover essential functions such as joining a meeting, using the chat feature/raising of hand, and managing audio/video settings. This will ensure that participants can fully engage in the sessions. |
|  | **WHEN and HOW MUCH**   8. Describe the number of times the intervention was delivered and over what period of time including the number of sessions, their schedule, and their duration, intensity or dose. | Page 12 | The oSR intervention will be delivered weekly over a period of 6 weeks. Each session will last between 1 hour and 1.5 hours, depending on the depth and length of the discussions. |
|  | **TAILORING**   9. If the intervention was planned to be personalised, titrated or adapted, then describe what, why, when, and how. | n/a | The intervention will not be personalised, titrated, or adapted for individual participants. |
|  | **MODIFICATIONS**   10. If the intervention was modified during the course of the study, describe the changes (what, why, when, and how). | n/a | As per the pre-registered OSF protocol, no changes have occurred. |
|  | **HOW WELL**   11. Planned: If intervention adherence or fidelity was assessed, describe how and by whom, and if any strategies were used to maintain or improve fidelity, describe them. | Page 14 | Intervention fidelity will be assessed either using the National Institute of Health (NIH) Behaviour Change Consortium or a similar model. |

** **Authors** - use N/A if an item is not applicable for the intervention being described. **Reviewers** – use ‘?’ if information about the element is not reported/not sufficiently reported.

† If the information is not provided in the primary paper, give details of where this information is available. This may include locations such as a published protocol or other published papers (provide citation details) or a website (provide the URL).

ǂ If completing the TIDieR checklist for a protocol, these items are not relevant to the protocol and cannot be described until the study is complete.

* We strongly recommend using this checklist in conjunction with the TIDieR guide (see *BMJ* 2014;348:g1687) which contains an explanation and elaboration for each item.

* The focus of TIDieR is on reporting details of the intervention elements (and where relevant, comparison elements) of a study. Other elements and methodological features of studies are covered by other reporting statements and checklists and have not been duplicated as part of the TIDieR checklist. When a **randomised trial** is being reported, the TIDieR checklist should be used in conjunction with the CONSORT statement (see [www.consort-statement.org](http://www.consort-statement.org)) as an extension of **Item 5 of the CONSORT 2010 Statement.** When a **clinical trial** **protocol** is being reported, the TIDieR checklist should be used in conjunction with the SPIRIT statement as an extension of **Item 11 of the SPIRIT 2013 Statement** (see [www.spirit-statement.org](http://www.spirit-statement.org)). For alternate study designs, TIDieR can be used in conjunction with the appropriate checklist for that study design (see [www.equator-network.org](http://www.equator-network.org)).
